# Is equipoise a useful concept to justify randomised controlled trials in the cultural context of Pakistan? A survey of clinicians in relation to a trial of talking therapy for young people who self-harm

**DOI:** 10.1186/s13063-023-07397-8

**Published:** 2023-08-08

**Authors:** Rakhshi Memon, Muqaddas Asif, Alexandra Pitman, Nasim Chaudhry, Nusrat Husain, Sarah J. L. Edwards

**Affiliations:** 1 University College London (UCL), London, UK; 2 Pakistan Institute of Living and Learning (PILL), Karachi, Pakistan; 3 Camden and Islington NHS Foundation Trust, London, UK; 4 University of Manchester (UoM), Manchester, UK

**Keywords:** Equipoise, Randomised clinical trials, Self-harm, Suicide, Culture, Evidence-base

## Abstract

**Background:**

Clinical equipoise, also defined as the uncertainty principle, is considered essential when recruiting subjects to a clinical trial. However, equipoise is threatened when clinicians are influenced by their own preferences. Little research has investigated equipoise in the context of trial recruitment.

**Methods:**

This cross-sectional survey sought clinicians’ views (operationalised as 11 statements relating to treatments offered in a trial of a psychological intervention for young people) about equipoise and individual treatment preferences in the context of moral justification for recruiting young people at risk of self-harm or suicide to a randomised controlled trial (RCT) to evaluate the Youth Culturally Adapted Manual Assisted Psychological Intervention (Y-CMAP) in Pakistan. We compared the views of clinicians involved in Y-CMAP RCT recruitment to those of a sample of clinicians not involved in trial recruitment but treating similar patients, comparing their sociodemographic characteristics and the proportions of those in each group agreeing with each statement.

**Results:**

There was a response rate of 96% (75/78). Findings showed that, during trial recruitment and before the RCT results were known, the majority of all responding clinicians (73.3%) considered Y-CMAP to be an effective treatment for young people at risk of self-harm or suicide. Although there was an acknowledgement of individual preferences for the intervention, there was near consensus (90%) on the need to conduct an RCT for reaching an evidence-based decision. However, there were no significant differences in the proportion of recruiting clinicians reporting a treatment preference for Y-CMAP than non-recruiting clinicians (31 (88.6%) versus 36 (90%), *p* = 0.566). A significantly higher proportion of non-recruiting clinicians (87.5%) as compared to (48.5%) in the trial (*p* = 0.000) stated that there may be other treatments that may be equally good for the patients, seemingly undermining a preference for the intervention. Those reporting a treatment preference also acknowledged that there was nothing on which this preference was based, however confident they felt about them, thus accepting clinical equipoise as ethical justification for conducting the RCT. There was a significant group difference in views that treatment overall is better as a result of young patients’ participation in the Y-CMAP trial (*p* = 0.015) (i.e. more clinicians not involved in the trial agreed with this statement). Similarly, more clinicians not involved in the trial agreed on the perceived availability of other treatment options that were good for young people at risk of self-harm (*p* < 0.05).

**Conclusions:**

The paper highlights that clinicians in Pakistan accept the notion of clinical equipoise as an ethical justification for patient participation in RCTs. The need for conducting RCTs to generate evidence base and to reduce bias was considered important by the clinical community.

## Background

There has been increasing academic and policy interest in the ethics of research over the past few decades. However, attention has largely been focussed on consent [1], and issues that have been neglected relate to ethics of evidence and clinical trials, and the role of other moral requirements such as equipoise and risk. Over 30 years ago, Benjamin Freedman introduced the concept of equipoise as “uncertainty or equal belief that there is equal balance between the two treatments in the trial and that one is equally effective than the other” [2].

Whilst this concept has since served as a foundation of research ethics, a debate has persisted over whether collective uncertainty amongst recruiting clinicians is sufficient or whether each individual clinician themselves needed to exist in a personal state of uncertainty. Freedman referred to ‘individual equipoise’ as when an individual clinician is uncertain and ‘collective or clinical equipoise’ as when a community of clinicians are uncertain. This is termed ‘clinical or collective equipoise’ and is often regarded as the most robust ethical principle on which conducting medical research and randomisation can be morally justified [3]. The ethical discourse on the relevance of clinical equipoise continues. It is interesting to note from Katz et al.’s [4] study that there is a marked divergence from the efficacy of a RCT, when participant recruitment is based on ‘individual equipoise’ and where clinicians’ selection enrolment is based on their own clinical preference. The study showed that there was more accuracy of trial results where ‘clinical equipoise’ exists [4]. The study discovered that selective enrolment disregarded the inclusion/exclusion criteria of the trial and introduced other clinically preferred factors of the clinician-researcher. Population-based selection purely grounded upon randomisation (clinical equipoise) resulted in more accurate trial results on the efficacy of the treatment. Appropriately executed RCTs are generally accepted to be the most dependable approach for comparing health technologies [5], whilst equipoise as the state of epistemic uncertainty is traditionally regarded as both the necessary and sufficient ethical condition stipulated by regulators such as Food and Drug Administration (FDA) Agency and National Institute of Clinical Excellence (NICE) to justify randomisation in clinical trials [5]. The scientific community continues to endorse clinical or collective equipoise as the most rigorous and ethical basis for undertaking randomised clinical trials based on the rationale that collective uncertainty accommodates the apparent fragility of individual equipoise [6]. However, acknowledging that there must be a scientific rationale behind an intervention to bother to trial it at all, there has recently been a resurgence of interest in equipoise due to new trial designs. The promise of innovative trial designs, such as response adaptive randomisation (RAR) [7], is that they offer a more ethical alternative conventional RCTs, in that they can use accumulating data and respond to threats to individual equipoise [8]. Deng et al. [9] propose that regulatory bodies should look at more innovative trial designs to cope with the problem of accumulating data and individual equipoise. For example, a response-adaptive cross-over trial design utilises the outcome data to decide whether to move any patient into the most effective treatment arm, so that the patient’s exposure to the inferior arm is reduced and as a consequence, minimises the risk to the patient whilst the clinical trial is underway. Some argue that this approach could be justified, even though there would be a lack of equipoise, if there would be a risk that the patient would be denied treatment by remaining in the inferior arm and where no other treatment exists [10]. A study by Legocki et al. [11] concluded that although clinical experts accept the advantages of adaptive clinical trials (ACT) such as having great potential to efficiently identify patients who will be helped most by specific treatments [12], however, it has to be pointed out that this is not without limitations, e.g. great vigilance on the part of investigators would be required, to ensure that the cross over process is adhered to, to avoid patients remaining on the non-responsive therapy and as a result coming to harm. A great risk to the trial itself would be the reduction of data at the latter part of the trial due to enhanced crossover or patient dropout. Additionally, it could be argued that there is potential for injustice in this approach as those patients recruited later would benefit from better treatment than those enrolled earlier in the trial.

As the controversy and debate continue, Hey et al. [7] state that “many clinical trials include procedures with some level of ‘net risk’ to participants”, meaning that the procedures are done purely for research purposes and hence do not promote participants’ “best clinical interests”. They propose “net risk” as an alternative to the concept of clinical equipoise.

Whichever conception is adopted, De Meulemeester et al. [13] in their cross-sectional analysis of published literature on RCTs conclude that equipoise, although widely accepted as scientific criteria and moral justification for conducting trials, was mentioned inconsistently, and was often misunderstood when reported. De Meulemeester et al. [13] argue that the utility of the concept of clinical equipoise as an ethical standard for justification of RCTs should therefore be challenged.

There is little analytic or empirical literature on the potential role of equipoise in justifying randomisation within two arms where the intervention is psychological intervention plus treatment as usual (TAU) versus TAU (which can include drug therapy). Indeed, this is the first published study of its type in the cultural and religious context of Pakistan.

Self-harm is a major risk factor for eventual suicide in Pakistan, and the prevention of self-harm is therefore a key focus for suicide prevention efforts [14]. Young people are especially at risk of suicide and self-harm [15]. For example, the prevalence of self-harm over 3 months in young people in India was 3.9 to 25.4% (wide range may denote uncertainty of scales) [14]. As such, young people with a history of self-harm are a priority group for interventions. There is growing evidence that psychological therapies, including those based upon cognitive behavioural therapy (CBT) principles, can help prevent further self-harm in those at risk, including young people [16]. However, whilst there have been robust RCTs establishing efficacy, the available evidence has been limited to Western and higher-income countries. Only one study with adolescents’ self-harm (12–18) from a low- and middle-income country (LMIC) is relevant to how we think about implementation methods of talking therapies in this age group [17].

## Findings in the context of other studies

Only seven published empirical studies have investigated equipoise during recruitment to RCTs. These were RCTs in clinical specialities such as oncology and surgery in UK hospital settings. None was from LMICs. Out of the seven, one concluded that there are practical challenges faced by clinicians when recruiting patients in RCTs, e.g. reported difficulties negotiating with equipoise due to its perceived frailty [18]. This study also reported that when recruiting patients’ clinicians can easily be influenced by their own personal beliefs and biases, which can have an impact on how equipoise is communicated to the patients [19]. This can result in disrupting equipoise, and the authors concluded it was important for clinicians to have training in communication skills [19]. Donovan et al. [20] stated that although doctors are comfortable with the concept of clinical equipoise as a general ethical framework for conducting RCTs, it becomes problematic for them when they are recruiting their own patients into a particular RCT. Donovan et al. [20] concluded that the application of equipoise varies from speciality to speciality. For example, surgeons would intuitively believe that surgery would be the best treatment option. Campbell et al. [21] reported that in many cases, most clinicians as researchers find it easier to explain the straightforward parts of the RCTs rather than the complex areas. This is quite concerning and raises the question as to how ‘informed’ is informed consent. Robinson et al. [22] in their studies concluded that descriptive information on equipoise and randomisation does not give participants of RCTs a better grasp of trial randomisation allocation and thus suggests that this may further complicate already complex trial information and may not aid informed decision-making [22].

There is growing literature to suggest that patients are unable to grasp the concept of equipoise [23]. These factors may have implications of personal bias being introduced in the consent process and consequently suggest the risk of disrupting equipoise. The other misconception by patients participating in RCTs is that although randomly allocated, their doctor would not allocate them to an inferior treatment and that there will already be a better treatment to which they would be allocated to, thus failing to accept that allocation would be at random [1, 23]. The situation in LMICs is even more challenging especially when results are available in one context but not in another. In order to achieve the UN Sustainable Development Goals, Implementation Research (IR) methodology requires consideration and understanding of the cultural and economic context in which clinicians weigh up their beliefs between the benefits and disadvantages of a certain treatment. Seward et al. [24] propose an enhanced approach to clinical equipoise, which they have termed *contextual equipoise.* This, they state, would ensure that the ‘do no harm’ principle of clinical equipoise is upheld when contextualised in real-world settings of less well-resourced countries.

Based on the above analysis and the importance of the issue, the current paper seeks to investigate whether clinicians recruiting patients to an RCT of Y-CMAP and those treating patients not involved in the trial had a personal preference for the intervention or whether they were in a state of ‘individual’ equipoise. For those who did express a preference for a particular arm, we were interested to find out which one they favoured, and whether they regarded participation in an RCT as scientifically and ethically important and necessary despite these personal preferences. The Y-CMAP Programme has been culturally adapted with permission from a self-help guide called “Life After Self-Harm” and “Cutting down: A CBT workbook for treating young people who self-harm” [25] to fit with the client’s problems and primarily utilises problem-solving, cognitive-behavioural assessments of self-harm and dialectical therapy strategies to bring about change.

## Objectives

The objectives of the survey were to (1) assess the preferences of clinicians involved in recruiting to a RCT and those not involved in the trial, regarding whether they preferred Y-CMAP plus TAU or TAU only for young patients at risk of self-harm and suicide, and (2) to determine the extent to which there is collective uncertainty in clinicians (i.e. equipoise) to justify conducting a RCT despite personal preferences.

## Methods

### Study design and setting

We conducted a cross-sectional survey of clinicians involved in the Y-CMAP at all five sites (Karachi, Lahore, Rawalpindi, Hyderabad and Peshawar Pakistan) of the trial (*n* = 35) and of clinicians not involved in the trial (*n* = 40) but practising in the same geographical areas. The total number of consented clinicians was *N* = 75.

Based on the Y-CMAP research teams’ experience with poor responses to online surveys, paper copies of the survey were physically taken to the clinicians. To recruit clinicians involved in the Y-CMAP trial, the Y-CMAP research team first elicited a ‘consent to contact’ form from the clinicians and then, for all those consenting to contact, the researchers gained informed consent by sending out invitation emails with information packs, including consent forms and scheduled a face-to-face meeting with clinicians who consented to complete the paper copies of the survey.

To recruit clinicians not involved in the Y-CMAP trial, we conducted convenience sampling of clinicians working in a mix of private and public healthcare settings within the Pakistan Institute of Living and Learning (PILL) network of research centres and contacts, including those in Karachi, Lahore, Rawalpindi, Hyderabad and Peshawar. These clinicians represented a similar mix of specialities (psychiatry, general practice, medical or surgical ward doctors, and other specialities). The process we followed was to contact the hospital administrator who provided the email addresses of clinicians. We shared study details with all of the clinicians but contacted only those who expressed interest in taking part. The consent procedure was the same as for clinicians involved in the trial.

The data were collected from May 2019 to December 2019, a period during which trial recruitment was ongoing and before the RCT results were known.

### Survey instrument

The questionnaire was adapted from a previously validated measure originally intended to assess therapeutic ‘misconception’ in patients when they consent to take part in research: the therapeutic misconception (TM) measure [26]. We adapted the wording of this self-administered measure to establish whether clinicians expressed a personal preference for either of the two treatments offered in the Y-CMAP RCT. Responses to each of the 11 statements were measured using Likert-type scales. To provide context for the survey questions, participants were provided with background information. This was provided in the form of previous relevant trial results on the effectiveness of interventions for adults who self-harm [16, 27].

The prototype questionnaire was piloted in January 2019 by four clinicians in Pakistan (two psychiatrists and two general practitioner (GPs) to test the contextual fitness [18] and face validity of the questionnaire. These four clinicians also took part in the main survey. Following advice from the PILL Advisory Group and supervisors regarding the risk of a central tendency [28], response options for the Likert-type scale were amended to remove the not sure option (reducing from five options to four) [26].

### Statistical analysis

We estimated that a minimum sample size of 50 clinicians was needed to detect significant group differences in proportions agreeing with statements [29]. It was expected that 30% of the clinician involved in the YCMAP trial and clinician not involved in the Y-CMAP trial up to 70%. For a standard significance level of 5% and to provide a power of 90%, 28 clinicians per group, 56 in total, were required. Allowing for an anticipated response rate of 90%, 25 participants in each arm were required and hence a total sample size of 50 to achieve 90% power. We compared the sociodemographic characteristics of those involved in recruiting to the trial, and those not involved in the trial, using chi-squared (*X*^2^) tests. Our hypotheses were as follows:

1. There are likely to be significant differences in clinicians involved in recruiting to the trial as compared to clinicians not involved in recruiting to the trial regarding treatment preferences (Y-CMAP plus TAU or TAU only) for young patients at risk of self-harm.
2. There is likely to be a collective uncertainty in clinicians (i.e. equipoise) in those involved in the trial and those not involved in the trial to justify conducting an RCT despite personal preferences.
3. We used the Pearson chi^2^ test to investigate the group differences in the proportion of those expressing different views relating to equipoise in a trial of an intervention for young people at risk of self-harm. Fisher exact test was used to determine the normality of data. SPSS version 23 was used for statistical analysis of data.

## Results

### Response

Initially, 78 clinicians were approached (38 involved in the trial, and 40 not involved) of whom 75 completed the survey questionnaire, representing a response rate of 96%. We recruited *n* = 35 clinicians involved in recruiting patients to the trial (46.7%) and *n* = 40 not involved in the trial (53.3%), representing a response of 92% (35/38) for clinicians involved in the trial group and of 100% for clinicians not involved in the trial (40/40).

### Demographic data

The total sample of 75 clinicians had a mean age of 30.88 years (SD = 9.28). Overall, 42 (56%) were male, and 33 (44%) were female. The mean number of years since graduation of clinicians from medical school was 6.4 (SD = 8.89) with a minimum of 1 year since graduation to a maximum of 35 years of graduation. Around 18.7% were GPs, 17.3% were psychiatrists, 4% were emergency department (ED) doctors, 22.7% were medical ward doctors, 12% were surgical ward doctors and 25.3% were doctors from other medical specialities (Table 1). A quarter (27.9%) of the sample reported having a relative or friend who had died by suicide, and 32.4% reported having a relative or friend who had attempted suicide. There were no significant group differences, comparing those involved in recruiting to the trial and those not involved, on any of these characteristics.

**Table 1:** Sociodemographic characteristics of participating clinicians (*N* = 75)

**Variable**	**Those involved in the trial (*n* = 35), *n* (%)**	**Those not involved in the trial (*n* = 40), *n* (%)**	**Total (*N* = 75), *n* (%)**	***p*-value**^†^
**Age**	30.65 (8.9)	31.07 (9.7)	30.8 (9.28)	.847
**Years since graduation from medical school**	5.80 (8.6)	6.92 (9.1)	6.4 (8.89)	.588
**Gender**				.225
Male	17 (40.5)	25 (59.5)	42 (56.0)	
Female	18 (54.5)	15 (45.5)	33 (44.0)	
**Medical speciality**				.380
GP	8 (57.1)	6 (42.9)	14 (18.7)	
Psychiatrist	8 (61.5)	5 (38.5)	13 (17.3)	
ED doctor	1 (33.3)	2 (66.7)	3 (4)	
Medical ward doctor	6 (35.3)	11 (64.7)	17 (22.7)	
Surgical ward doctor	2 (22.2)	7 (77.8)	9 (12)	
Other medical specialities	10 (52.6)	9 (47.4)	19 (25.3)	
**Do you know anyone amongst your friends or family who has died by suicide?**				
Yes	10 (14.7)	9 (13.2)	19 (27.9)	.787
No	24 (35.3)	25 (36.8)	49 (72.1)	
**Do you know anyone amongst your friends or family who has attempted suicide?**				
Yes	11 (16.2)	11 (16.2)	22 (32.4)	1.00
No	23 (33.8)	23 (33.8)	46 (67.6)	

For continuous variables (i.e. age and years since graduation from medical school), the mean and standard deviations are reported

*n* Number, *%* Percentage, *p-value* level of significance, *GP* General practitioner, *ED* Emergency department

### Missing data

There were no missing data on any variable.

### Comparison of views by involvement in the trial

The study found that there were no significant group differences (*p* > 0.05) in the proportions agreeing with 9 out of 11 statements (see Table 2 statement no. 1, 2, 5, 6, 7, 8, 9, 10, and 11). The majority of clinicians involved in the trial (60%) and half of the clinicians not involved in the trial (50%) disagreed with the statement that young people at risk of self-harm who are receiving Y-CMAP plus TAU in the trial may not do as well as they would on TAU alone (*p* > 0.05) (Table 2 statement no. 1). A high proportion of clinicians in both groups agreed that young people at risk of self-harm and suicide who are receiving Y-CMAP plus TAU may do better than TAU alone (Table 2 statement no. 2). Similarly, a significantly high percentage of clinicians involved in the trial (82.9%) and those not involved in the trial (65.0%) disagreed with the idea that Y-CMAP plus TAU may not be as effective as any TAU (*p* > 0.05) (Table 2 statement no. 5).

**Table 2:** Comparison of the proportions of clinicians expressing agreement with survey statements

Variable	Those involved in the trial (*n* = 35), *n* (%)	Those not involved in the trial (*n* = 40), *n* (%)	Total (*N* = 75), *n* (%)	*p*
**1. Young patients at risk of self-harm who are receiving Y-CMAP plus TAU in the trial may not do as well as they would on TAU alone**				
Agree	14 (40.0)	20 (50.0)	34 (45.3)	.263
Disagree	21 (60.0)	20 (50.0)	41 (54.7)	
**2. Young patients receiving Y-CMAP plus TAU may do better than they would on TAU alone**				
Agree	34 (97.1)	37 (92.5)	71 (94.7)	.360
Disagree	1 (2.9)	3 (7.5)	4 (5.3)	
**3. Treatment overall is better as a result of young patients’ participation in the Y-CMAP trial**				
Agree	22 (62.9)	36 (90.0)	58 (77.3)	.015
Disagree	13 (37.1)	4 (10.0)	12 (22.7)	
**4. There are other treatments outside this study which might be just as good for them**				
Agree	17 (48.6)	35 (87.5)	52 (69.3)	.000
Disagree	18 (51.4)	5 (12.5)	23 (30.7)	
**5. Y-CMAP plus TAU may not be any more effective than any TAU**				
Agree	6 (17.1)	14 (35.0)	20 (26.7)	.068
Disagree	29 (82.9)	26 (65.0)	55 (73.3)	
**6. I think that the Y-CMAP plus TAU is preferable to TAU alone for young patients**				
Agree	31 (88.6)	36 (90.0)	67 (89.3)	.566
Disagree	4 (11.4)	4 (10.0)	8 (10.7)	
**7. There is nothing on which to base a personal preference for Y-CMAP plus TAU ahead of TAU alone for young patients**				
Agree	19 (54.3)	30 (75.0)	49 (65.3)	.051
Disagree	16 (45.7)	10 (25.0)	26 (34.7)	
**8. Personal preference for Y-CMAP plus TAU is permissible despite collective uncertainty amongst the scientific profession**				
Agree	30 (85.7)	32 (80.0)	62 (82.7)	.367
Disagree	5 (14.3)	8 (20.0)	13 (17.3)	
**8a. I am confident in this preference**				
Agree	33 (94.3)	37 (92.5)	70 (93.3)	.564
Disagree	2 (5.7)	3 (7.5)	5 (6.7)	
**9. A personal preference could be based on factors independent of a clinical judgement of relative efficacy**				
Agree	32 (91.4)	34 (85.0)	66 (88.0)	.312
Disagree	3 (8.6)	6 (15.0)	9 (12.0)	
**10. The randomised trial of Y-CMAP plus TAU and TAU alone is clinically needed despite any individual preferences for Y-CMAP plus TAU or for TAU alone**				
Agree	32 (91.4)	35 (87.5)	67 (89.3)	.434
Disagree	3 (8.6)	5 (12.5)	8 (10.7)	
**11. The trial of Y-CMAP plus TAU and TAU alone should be sufficient to decide whether to offer Y-CMAP as a standard treatment for young people at risk of self-harm**				
Agree	26 (74.3)	30 (75.0)	56 (74.7)	.576
Disagree	9 (25.7)	10 (25.0)	19 (25.3)	

*n* number of clinicians in each group, *%* percentage, *p* significance level

We found no significant group differences in the proportions reporting a preference for Y-CMAP plus TAU for young patients over TAU alone (over 85% in each group; *p* > 0.05; statement 6 in Table 2), or in the proportions agreeing that there is nothing on which to base a personal preference for Y-CMAP plus TAU over TAU alone for young patients (54.3% vs 75.0%, *p* > 0.05) (Table 2 statement no. 7). Consistently, there were no significant differences found in reported personal preferences for Y-CMAP plus TAU despite collective uncertainty amongst the scientific profession (*p* = 0.367) and their reported confidence in this preference (statement no 8, and 8a in Table 2) (*p* = 0.564). Almost equal percentages in both groups (more than 90%) agreed that they were confident in this preference. More than 80% of clinicians in both groups agreed that possessing a personal preference for Y-CMAP plus TAU is morally permissible despite collective uncertainty amongst the scientific profession.

Nearly 91.4% of clinicians involved in the trial agreed that personal preference could be based on factors independent of a clinical judgement of relative efficacy (statement no. 9) compared with 85% of those not involved in the trial showing a non-significant difference (*p* > 0.05). Similarly, although there were no significant differences (*p* > 0.05), the majority of the clinicians involved in the trial (91.4%) and a nearly equal but slightly lower percentage of clinicians not involved in the trial (87.5%) agreed that randomised trial of Y-CMAP plus TAU and TAU alone is clinically needed despite any individual preferences for Y-CMAP plus TAU or for TAU alone (statement no. 10).

There were no significant differences found in the statement that the trial of Y-CMAP plus TAU and TAU alone should be sufficient to decide whether to offer Y-CMAP as a standard treatment for young people at risk of self-harm. Almost equal percentages of both groups (nearly 75%) agreed that the trial of Y-CMAP plus TAU and TAU alone should be sufficient to decide whether to offer Y-CMAP as a standard treatment for young people at risk of self-harm (statement no. 11).

Specific statements for which we found significant group differences were the following:

- We found that a significantly greater proportion of clinicians not involved in the trial agreeing with the statement that treatment overall is better as a result of young patients’ participation in the Y-CMAP trial (62.9% vs 90%; *p* = 0.015; statement no. 3).
- Regarding the perceived availability of other treatment options for young people at risk of self-harm, a significantly greater proportion of clinicians not involved in the trial agreed with the statement that there are other treatments which might be just as good for young people (87.5% versus 48.6%; *p* < 0.05; statement 4 in Table 2). However, a significant majority of clinicians involved in the trial (51.4%) as compared to those not involved in the trial (12.5%) disagreed with this statement (*p* < 0.05) (Table 2 statement no. 4).

There was an interesting set of apparently incoherent responses from clinicians treating patients, not involved in the trial. For example, those clinicians treating patients not involved in the trial simultaneously accepted that Y-CMAP plus TAU *may* be more beneficial than TAU alone (statement 2) and that, other treatment options *could* be equally beneficial to Y-CMAP (statement 5).

## Discussion

The data from the survey helped to inform the issue of which conception of equipoise is morally appropriate for RCTs such as Y-CMAP in Pakistan. A majority (89.3%) of clinicians both involved in the trial and those not involved in the trial preferred Y-CMAP plus TAU rather than TAU alone for young people at risk of self-harm and suicide. TAU in Pakistan may represent no care, other psychological or pharmacological therapies, or spiritual guidance and religious therapy.

It is interesting to note that, despite the fact that clinicians believed that Y-CMAP plus TAU would be an effective treatment for patients at risk of self-harm and suicide, a higher percentage of clinicians not involved in Y-CMAP stated that there may be other treatments that may be equally good for the patients. This is consistent with findings reported from an Iranian sample of medical students, nurses and the general public simulating membership of an ethics committee [17] suggesting “clinical or collective equipoise would rarely …. equally divide preferences amongst individual clinicians to reflect the RCT to which they are collectively recruiting patients” [30].

More than half of the clinicians from both groups agreed that randomised trial of Y-CMAP plus TAU and TAU alone is clinically needed despite any individual preferences and that this trial should be sufficient to decide whether to offer Y-CMAP as a standard treatment for young people at risk of self-harm and suicide. Irrespective of personal experiences of the suicide attempt or suicide of relatives or friends, both groups agreed that randomisation was ethically and morally justified in a trial to gain an objective view. This is in contrast with the findings of the Iranian sample described above.

The response to the two optional questions was 100%. The survey data from these voluntary questions revealed a high prevalence of self-harm and suicide in health professionals’ family and friends (27.9%) knew someone who had died with suicide and (32.4%) who knew someone, who had attempted suicide. This data suggests individual bias in qualitative results. Also, individual clinicians cited real examples, which suggest treatment preferences to be stronger. Thus, making the stance towards clinical equipoise as justification for RCTs even more robust.

### Clinical, research and policy implications

Therapies that work in Western countries cannot necessarily be implemented in their current form, but need to be adapted to account for cultural differences (differences in the understanding of mental health and self-harm, e.g. stoicism and fatalism). As well as adapting the intervention itself, Pakistan is a challenging context in which to evaluate treatments through RCTs.

Johnson et al. [30] suggest that it may be problematic to justify a trial which is based on a pre-existing trial that is deemed to be efficacious. Applying this to the Y-CMAP paradigm, awareness of the findings of the CMAP trial in adults may have prejudiced clinicians’ beliefs on the relative benefits and disadvantages of CMAP in young people. On the contrary, even though there was acceptance by some clinicians of individual treatment preferences, it is noticeable that the proportion of clinicians involved in the trial was lower as compared to clinicians not involved in the trial (statement 7). Nonetheless, there was consensus on the need to conduct an RCT for reaching an ‘evidence-based’ decision. The reason for this was primarily to reduce bias, suggesting that there seems to be enough evidence of uncertainty and the existence of clinical equipoise as ethical justification of conducting the randomised clinical trial.

Although there were no statistically significant group differences amongst clinicians (statement 9), more clinicians involved in recruiting to the trial appreciated that personal preference for the intervention could be based on factors independent of prior belief in its relative clinical efficacy.

A key point to highlight here is that TAU in Pakistan may mean no treatment at all, and this might influence clinicians’ personal beliefs regarding the benefits of recruiting their patients to a RCT as a means for them to gain access to a potentially beneficial intervention. Interestingly, it was not clear what were the treatment options that the clinicians not involved in the trial considered to exist in terms of those equally good as Y-CMAP. Rather than receiving nothing at all, it was possible that this group anticipated young people being offered other talking therapies or access to spiritual guidance. More work is needed to show how much faith clinicians hold in the concept of evidence-based medicine and what they consider to be the ethical conditions under which RCTs are undertaken.

### Strengths and limitations

As far as we are aware, this is the first survey of its kind conducted in a LMIC setting, in the context of very few similar studies conducted in high-income countries, and findings are of interest to ethicists, trialists and clinicians in other countries.

One strength of this study was that we sampled pragmatically from clinicians involved in a trial, comparing them to those not involved, providing a valuable comparison. Our questionnaire was designed to check the internal consistency of responses, by using a set of similar questions. Our pilot suggested that our questionnaire had good face validity. The inconsistencies in some views from clinicians involved in recruiting patients in the trial may be explained by those clinicians having interpreted these statements differently due to a greater familiarity with this specific RCT (or others) restricting their definition of a TAU to those having been scientifically evaluated. Alternatively, the way some of the questions were phrased may have been interpreted as possibilities rather than expectations of relative efficacy or a request for logical ranking from which we have inferred preferences for the intervention.

We acknowledge the potential for social desirability bias in the responses. Although concepts were defined at the beginning of the questionnaire and in the accompanying letter, it was also possible that some clinicians responding had a lack of understanding of the concept of equipoise. As this was the first study of this kind, we accept some limitations in questionnaire design and response options that could be modified in future research. As this was a self-reported questionnaire, we cannot be sure how clinicians interpreted these as self-reported questionnaires are subjective.

Lastly, we did not ask about how accumulating data within the trial could disturb equipoise and render the trial unethical. However, we did ask whether positive RCT results would be sufficient for Y-CMAP to become a standard option, which the majority in both groups agreed with.

## Conclusion

Clinicians in Pakistan, whether they are recruiting for a trial such as Y-CMAP or not, accepted the notion of clinical equipoise as a moral justification for RCTs. The majority of clinicians involved in recruiting patients to the Y-CMAP trial, and the majority of those not involved in recruiting patients to the trial, considered Y-CMAP as an effective treatment for young patients at risk of self-harm or suicide even before the trial. However, the need to conduct the RCT to generate evidence base and reduce bias was considered important.

## Data Availability

The anonymised dataset will be available from the corresponding author upon reasonable request.
